# GEROTRANSCENDENCE INTERVENTIONS FOR CAREGIVERS: A SCOPING REVIEW

**DOI:** 10.1093/geroni/igad104.2177

**Published:** 2023-12-21

**Authors:** Oscar Ribeiro, Lia Araujo, Taiane Abreu, Laetitia Teixeira

**Affiliations:** University of Aveiro, Aveiro, Aveiro, Portugal; Cintesis, Porto, Porto, Portugal; ICBAS, Porto, Porto, Portugal; School of Medicine and Biomedical Sciences, University of Porto, Porto, Porto, Portugal

## Abstract

According to Tornstam, gerotranscendence is a psychosocial adaptive theory of aging that postulates over a gradual mindset shift that occurs from middle age and progresses during old age, with individuals becoming less materialistic and more transcendental in actions and thoughts. Despite being a natural process, interventions based on gerotranscendence have been associated with improvements in well-being and are starting to receive attention among professionals working with older adults. This study aims to map interventions based on gerotranscendence targeting older adults’ caregivers and summarize their main outcomes. Six databases were searched, and three final studies were selected and analyzed based on the Template for Intervention Description and Replication (TIDieR). All studies took place in nursing homes and long-term care (LTC) facilities with staff members. Two studies presented a long-term intervention with a qualitative approach, while one presented a short-term intervention with a quasi-experimental approach. All interventions had a moment of group instruction about the gerotranscendence theory, in which participants were informed about gerotranscendence signs that could be recognized and/or promoted in older adults. All three studies reported positive outcomes suggesting that informing and training caregivers on gerotranscendence can contribute to a higher quality of care and to an improvement of the caregiving relationship.

